# Case report: A patient with EGFR L861Q positive adenosquamous lung carcinoma transforming into large cell neuroendocrine cancer after treatment with Almonertinib

**DOI:** 10.3389/fonc.2025.1453066

**Published:** 2025-02-18

**Authors:** Kele Cheng, Yong Zhu, Ran Sang, Zhongsheng Kuang, Yang Cao

**Affiliations:** ^1^ The First Clinical School of Guangzhou University of Chinese Medicine, Guangzhou, China; ^2^ Department of Oncology Center, the First Affiliated Hospital of Guangzhou University of Chinese Medicine, Guangzhou, China

**Keywords:** Almonertinib, EGFR, mutation, neuroendocrine, large cell neuroendocrine carcinoma, LCNEC, adenosquamous carcinoma

## Abstract

Almonertinib, a third-generation epidermal growth factor receptor tyrosine kinase inhibitor, is selective for both epidermal growth factor receptor tyrosine kinase inhibitor–sensitizing and T790M resistance mutations. However, resistance to the third-generation EGFR-TKIs is still inevitable. Econdary EGFR mutations, and bypass pathway activation have been reported with Almonertinib therapy. This article presents a rare case report of a patient with EGFR L861Q positive adenosquamous lung carcinoma who transformed into large cell neuroendocrine carcinoma following treatment with Almonertinib. The patient exhibited disease progression 8 months after initiating Almonertinib treatment, and a blood genetic test revealed mutations in EGFR L861Q and EGFR L858R. A subsequent lung biopsy after progression confirmed the diagnosis of large cell neuroendocrine carcinoma, and subsequently treatment with cisplatin and etoposide was effective. Transformation into neuroendocrine carcinoma is one of the mechanisms behind resistance to Almonertinib in adenosquamous lung carcinoma. EGFR mutations may persist even after transformation into neuroendocrine carcinoma. For non-small cell lung cancer patients undergoing Almonertinib therapy, this case report emphasizes the importance of performing a timely pathological biopsy upon the emergence of resistance.

## 1 Introduction

Adenosquamous carcinoma (ASC) of the lung is a relatively rare subtype of non-small cell lung cancer (NSCLC), accounting for only 2%-3% of all lung cancers (1, 2). Studies have shown that ASC is more commonly found in male patients, and a history of smoking has been confirmed as a high-risk factor for ASC (1, 3–6). Known mutations in ASC include those in EGFR, ERBB2, KRAS, BRAF, PIK3CA, RET, ALK, and others (7). This article reports a rare case of a patient diagnosed with EGFR positive adenosquamous lung carcinoma, who experienced disease progression following treatment with Almonertinib. Repeat biopsies revealed that the resistance mechanism was transformation into large cell neuroendocrine carcinoma (LCNEC). Chemotherapy treatment was administered in response to this finding, leading to successful disease control in the patient.

## 2 Case reports

**Figure 1:**
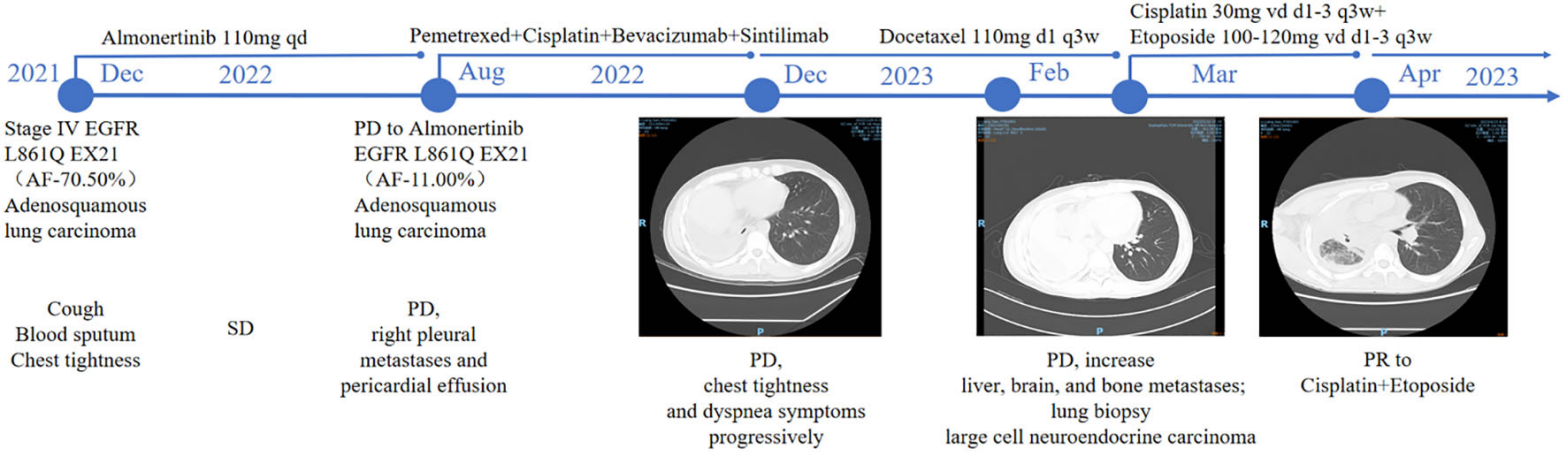
Timeline of the clinical course of the patient. Arrows indicate the link between the clinical event and the date of biopsy time point.

**Figure 2:**
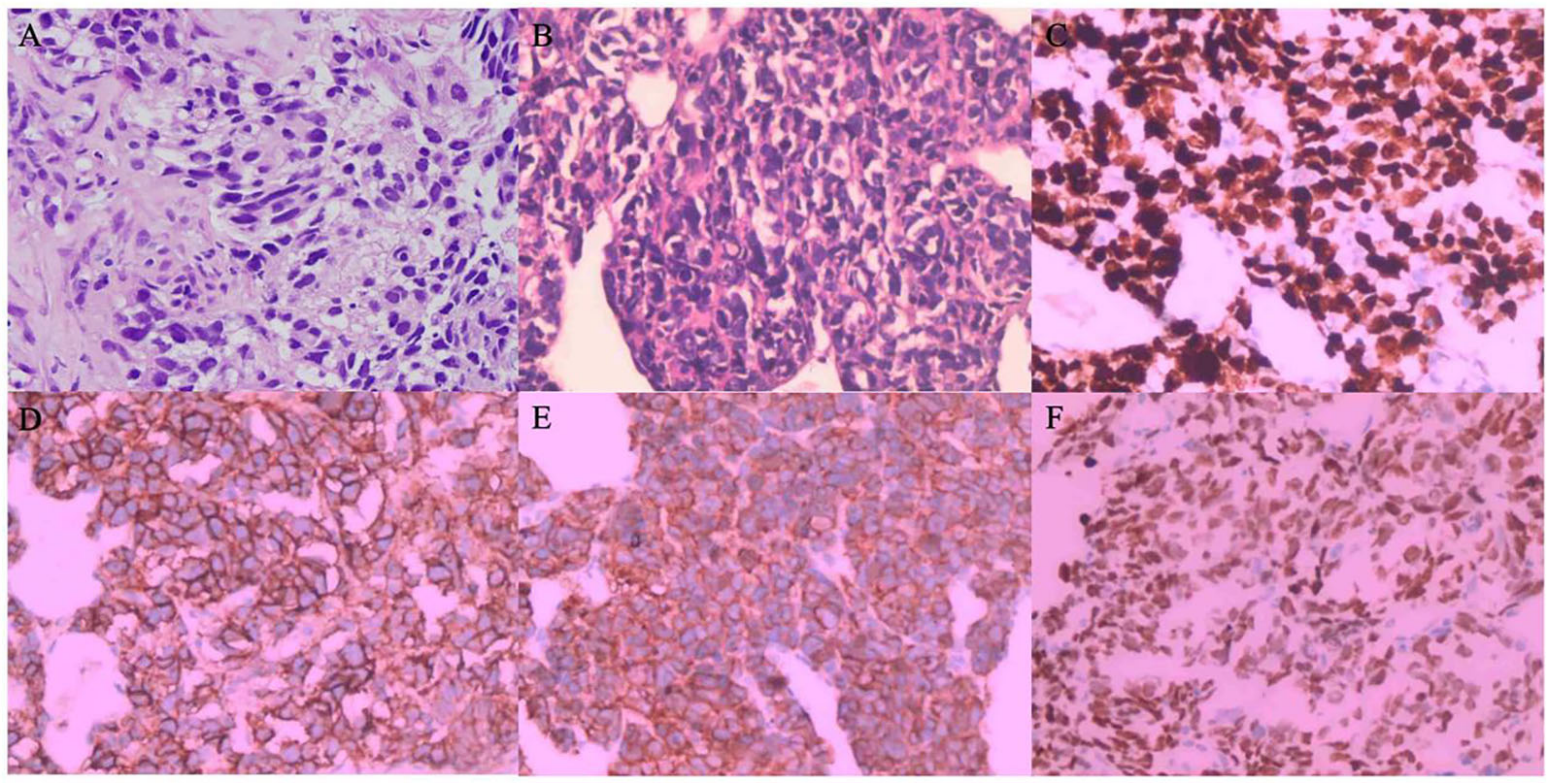
**(A–E)** Hematoxylin & Eosin Staining and Immunohistochemical Staining - **(A)** Hematoxylin and eosin-stained section (200x, December 2021) depicting focal solid nests of tumor cells diagnostic of adenosquamous carcinoma. **(B)** Hematoxylin and eosin staining reveals cellular architecture (200x, February 2023); **(C)** High Ki-67 index (>95%) suggests aggressive proliferation; **(D)** CD56 positivity indicates neuroendocrine differentiation; **(E)** Synaptophysin positivity and **(F)** TTF-1 expression confirm neuroendocrine phenotype and lung origin, consistent with a transition to large cell neuroendocrine carcinoma. **(B)** Hematoxylin and eosin staining reveals cellular architecture depicting focal solid nests with the infiltrative growth. Tumor cells had large dark irregular nuclei with atypical and numerous mitotic figures diagnosing of large cell neuroendocrine carcinoma (200x, February 2023); **(D)** CD56 positivity suggests neuroendocrine differentiation; **(E)** Synaptophysin immunoreactivity further supports neuroendocrine features; **(F)** TTF-1 expression, consistent with lung origin, all indicative of a transition to large cell neuroendocrine carcinoma. **(D)** CD56 immunostaining and **(E)** highlighting positive expression (200x, February 2023) characteristic of large cell neuroendocrine carcinoma.

The patient is a 43-year-old Han Chinese male with no history of smoking. The timeline of the clinical course of the patient was presented in **Figure 1**. In November 2021, he presented with symptoms of cough, hemoptysis, and chest tightness. A CT scan revealed a cancer of the right upper lobe, with mediastinal right hilar and right pleural metastases. In December 2021, biopsies of the lung mass and pleura were conducted, with the pathology indicating adenosquamous carcinoma. Immunohistochemistry revealed nests of carcinoma cells with focal solid areas, CK(+), CK7(+), CK5/6(+), P63(+), P40(+), weakly positive for TTF-1, and S100 (-) (**Figure 2A**). The clinical stage was cT4N1M1a,IVa. Tissue genetic testing results showed an EGFR L861Q mutation (AF-70.50%) and a PTEN mutation (AF-67.20%). The patient subsequently started oral Almonertinib 110mg qd and regular monitoring showed a stable disease (SD) response. In August 2022, a follow-up CT indicated right pleural metastases and pericardial effusion, with the treatment response evaluated as progressive disease (PD). As the patient refused repeat biopsy, blood genetic testing was performed, revealing EGFR L861Q mutation (AF-11.00%), EGFR L858M mutation (AF-11.94%), and other mutations including RB1 (AF-9.20%), TP53 (AF-12.97%), PTEN (AF-12.51%), and SMARCA4 (AF-0.96%). The patient then received three cycles of pemetrexed, cisplatin, bevacizumab, and sintilimab treatments. In December 2022, a CT scan showed occlusion of the right main bronchus, atelectasis of the right lung, and metastases to the right hilar and mediastinal lymph nodes. There was invasion into the right pleura and pericardium, with increased right pleural and pericardial effusions. In February 2023, a CT scan showed a further increase in pleural and pericardial effusion, along with liver, brain, and bone metastases. From August 2022 to February 2023, the patient’s chest tightness and dyspnea symptoms progressively worsened. Due to a significant rise in neuron-specific enolase (NSE), the patient underwent another lung biopsy in February 2023, which revealed large cell neuroendocrine carcinoma. Immunohistochemistry showed P40 (-) and P63 (-), TTF-1(+), CK7(-), CD56(+), Syn(+), and CgA(+), negative for ALK (D5F3) and PD-L1 (TPS: 0%), with Ki-67(95%+) (**Figures 2B–E**). The findings suggested a transformation from ASC to LCNEC following targeted therapy. Following this, the patient received two cycles of an “EP” regimen (cisplatin + etoposide) chemotherapy in March and April 2023. After chemotherapy, the follow-up CT scan showed a partial response (PR), and the patient’s symptoms significantly improved.

## 3 Discussion

To our knowledge, this is the first reported case of an ASC patient transforming into LCNEC following treatment with Almonertinib. ASC is an infrequent subtype of NSCLC, defined by the presence of admixed glandular and squamous cell components, each comprising more than 10% of the tumor. ASC generally exhibits more aggressive behavior and is associated with a poorer prognosis compared to cancers of solely adenocarcinoma or squamous cell histology (6, 8). Recent studies suggest that both glandular and squamous components in ASC likely originate from a single clonal event, followed by transdifferentiation from adenocarcinoma to squamous cell carcinoma (9). The frequency of EGFR mutations in ASC is comparable to that in pulmonary adenocarcinoma (10, 11), and patients with EGFR-mutant ASC respond favorably to EGFR-TKI therapies (12).

Almonertinib is the first third-generation EGFR-TKI drug developed independently in China, designed to overcome the common T790M resistance mutation. The AIM study, reported at the ESMO Asia 2022, enrolled advanced non-squamous NSCLC patients with uncommon EGFR mutations and indicated that Almonertinib was effective for first-line treatment of NSCLC patients with uncommon EGFR mutations other than EGFR ex20ins (13). Almonertinib demonstrated better efficacy in patients with G719X, L861Q, and S768I mutations compared to other genotypes. The results of the AENEAS study highlighted that in first-line treatment of locally advanced or metastatic NSCLC patients with EGFR mutations, Almonertinib significantly prolonged median progression-free survival (PFS) (19.3 months versus 9.9 months) and duration of response (DOR) (18.1 months versus 8.3 months), with a more favorable safety profile compared to gefitinib (14).

In this present case, the patient was diagnosed with EGFR L861Q positive ASC and treated with Almonertinib. However, after 8 months, disease progression was noted. Despite the extended survival benefits that third-generation EGFR-TKIs have brought to NSCLC patients with EGFR mutations, the problem of resistance remains unavoidable. In phase II clinical trials, resistance mechanisms to second-line Almonertinib predominantly involved secondary EGFR mutations (such as cis C797S and L718Q mutations) as well as bypass activation (including mutations in PIK3CA, JAK2, BRAF, KRAS, HER2 amplification, and FGFR3-TACC3 fusion) (15). After disease progression, our patient underwent repeat blood genetic testing, which identified mutations in EGFR L861Q, EGFR L858M, RB1, TP53, PTEN, and SMARCA4. With the repeat biopsy suggesting LCNEC, we can ascertain that the resistance mechanism was transformation into a neuroendocrine tumor rather than secondary EGFR mutations or bypass activation.

LCNEC and SCLC both belong to high-grade neuroendocrine carcinomas, sharing similar clinical and genomic features, suggesting a common pathway for transformation. In studies by Ferre (16) and Marcoux (17), median times from initiation of treatment to phenotypic transformation for EGFR-mutant adenocarcinomas were 16 and 17.8 months, respectively. Possible reasons for transformation from adenocarcinoma to SCLC include: 1. coexistence of adenocarcinoma and SCLC at tumor onset, with SCLC eventually superseding other histological types following TKI treatment; 2. besides originating from pulmonary neuroendocrine cells (18), SCLC may arise from other lung epithelial cells (19), with type II pneumocytes having the potential to differentiate into both histologies, possibly giving rise to both EGFR-mutant adenocarcinoma and SCLC (20); 3. inactivation of TP53 and RB1 genes (20–22), which are commonly lost in SCLC, plays an inducing role in the occurrence of SCLC (23). Additionally, loss of RB family members P107 or P130, amplification of the MYC family, alterations in the PTEN pathway, and high expression of BCL-2 are associated with SCLC cell growth, proliferation, and survival (24). Changes in the lung stroma and immune microenvironment might also contribute to occurrence of SCLC (25). Other genetic pathways that are probably involved in the histopathological transformation are NOTCH and ASCL1 (26). ASCL1 is targeted by NOTCH signaling (27) and research suggested that one inactivating NOTCH mutation was sufficient to induce neuroendocrine differentiation from nonneuroendocrine tumor cells or tumor precursors (28). While research on the transformation mechanism of LCNEC is scarce, given the clinical and genomic similarities between LCNEC and SCLC (29, 30), we speculate that the pathways of transformation to LCNEC are similar to those of SCLC, warranting further studies. Moreover, while the mechanism of adenocarcinoma transformation is relatively well understood, the mechanism by which ASC transforms into a neuroendocrine carcinoma following EGFR-TKI treatment remains to be further explored.

Studies have indicated that NSCLC patients with concurrent EGFR/RB1/TP53 mutations are more prone to transform into SCLC (31), especially those with a high frequency of AID/APOBEC mutations and genomic amplifications. Our patient’s secondary genetic testing revealed an EGFR/RB1/TP53 triplet mutation set. Regrettably, after developing resistance to EGFR-TKI therapy, he refused repeat biopsy and instead received three cycles of combined therapy with pemetrexed, cisplatin, bevacizumab, and sintilimab. Owing to ongoing tumor progression and significantly elevated NSE levels, we performed a lung biopsy 6 months post resistance to EGFR-TKI therapy, which confirmed a diagnosis of LCNEC. Treatment for LCNEC currently centers around chemotherapy, with the optimal regimen still debated; often, SCLC chemotherapy protocols are referenced. Thus, we opted for the EP regimen, and after two cycles, a follow-up CT scan indicated overall tumor shrinkage with a PR evaluation, and the patient’s symptoms substantially relieved, confirming the efficacy of the treatment.

## 4 Conclusion

Our case demonstrates that transformation into a neuroendocrine tumor is one of the mechanisms of acquired resistance to Almonertinib, and that the retention of EGFR mutations may also occur in LCNEC. Identifying the histological diagnosis and driver mutations is important in deciding treatment options. Therefore, we recommend timely repeat biopsies for patients who develop resistance to EGFR-TKI therapy, particularly those with concurrent EGFR/RB1/TP53 mutations in NSCLC, in order to obtain the best treatment strategies to further extend patient survival and improve quality of life. Chemotherapy can be considered for the EGFR/RB1/TP53 mutation triad. Chemotherapy remains effective for neuroendocrine carcinoma post-tumor transformation. We look forward to the new development of molecular detection methods for early identification of transformation risks in the future.

## Data Availability

The original contributions presented in the study are included in the article/supplementary material. Further inquiries can be directed to the corresponding author.
